# Supplementary material for: Prevalence of unrecognized depression and associated factors among medical outpatient department attendees; a cross sectional study
Source: PLoS One. 2021 Dec 21;16(12):e0261064. doi: 10.1371/journal.pone.0261064 (PMC8691632; doi:10.1371/journal.pone.0261064)
Supplement: Supplementary file 1 [file pone.0261064.s001.docx]

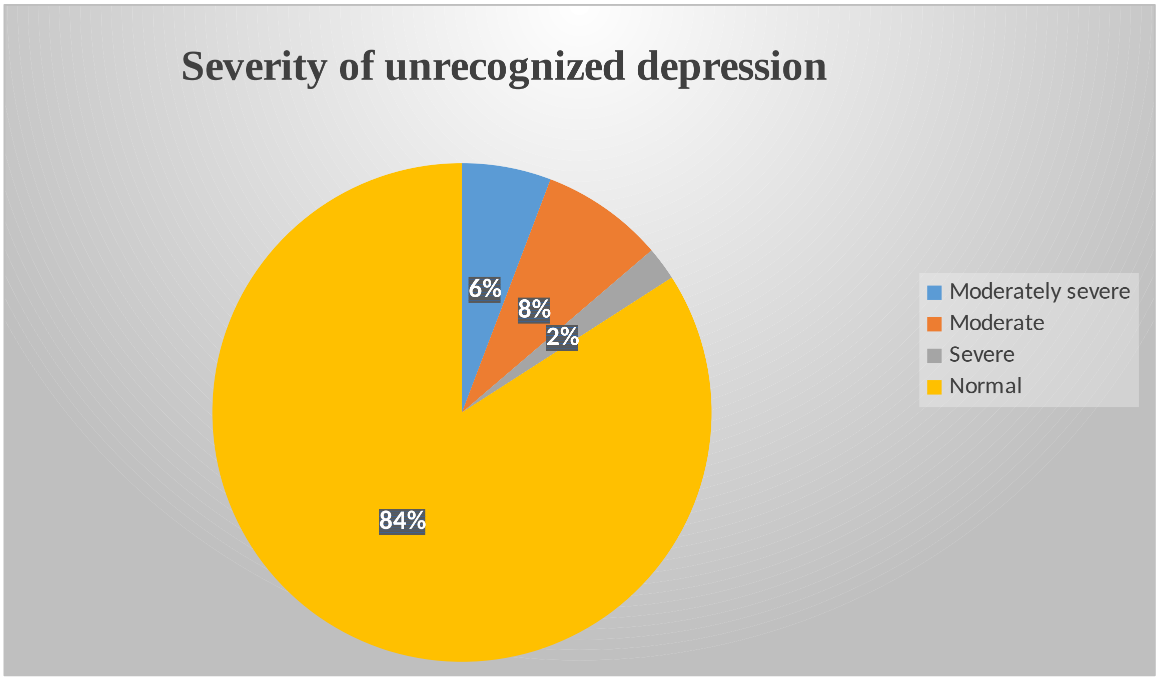
Severity of unrecognized depression
6%
8%
2%
84%
Moderately severe
Moderate
Severe
Normal
